# A Peep behind the Scenes

**Published:** 1913-11-08

**Authors:** 


### EDITOR'S LETTER-BOX.

### "A Peep Behind the Scenes."

*To the Editor of* The Hospital.

Sir,—A few days back I went into a chemist's shop in one of our large cities, and while the assistant was obtaining the drug which had been asked for I had the opportunity of seeing behind the counter. There I observed the bottle boy "cleaning" the pill mould, which he did by spitting upon it and then vigorously polishing with a duster!—Yours faithfully, Ex-Chaplain.

